# Our first 5 years: advancing the health and wellbeing of people in the Western Pacific region

**DOI:** 10.1016/j.lanwpc.2025.101680

**Published:** 2025-08-29

**Authors:** The Lancet Regional Health – Western Pacific

*The Lancet Regional Health—Western Pacific* celebrates its fifth anniversary this month. Since our inaugural issue was published online in August, 2020, our team has grown from one member to five, based in Beijing, Shanghai, and Sydney. We have launched regular podcasts where our authors share their work and discuss its impact and significance. Importantly, our content has evolved from a focus on monitoring and mitigating the COVID-19 pandemic to addressing a broader range of public health challenges and critical clinical questions in the Western Pacific region.

Our 5-year journey underscores that health needs are diverse across communities and countries; similar health issues can be interpreted and managed differently depending on the local health-care systems. For instance, chronic pain management tends to be more conservative in Asia compared with European and America. Nonetheless, many widely discussed health topics affecting vulnerable populations still receive little attention in the Asia–Pacific region due to stigma, cultural norms, negligence, or lack of awareness. We are aptly placed to serve as a platform that amplifies these needs and voices from the Western Pacific, highlighting topics such as sexual health for Chinese older adults, migrant health in Japan, racism and health in South Korea.

In addition, we monitor research trends to accurately reflect health needs and to inform health priorities in the region. Much of our current understanding of public health interventions and disease management is based on research from Europe and North America; however, there is a growing recognition of the value of local and indigenous knowledge in addressing the current health challenges. In the *Cancer in First Nations Australians Series* published in this issue, Sandra C Thompson and colleagues discussed disparities in cancer care for First Nations Australians and highlighted the capabilities within First Nations communities to develop culturally safe and accessible care through co-design and Indigenous leadership. Health advocacy remains largely conceptualised and led by researchers and clinicians, with patient perspectives often missing in the Western Pacific region. Also in this issue, Yuankai Shi and colleagues discuss the current situation and challenges of patient-reported outcomes in oncology clinical trials in China. The meaningful engagement of individuals with lived experience and organisations like Common Sense Oncology in the Western Pacific region are needed to advance patient-centred care in the region.

Integrating local and patient knowledge into health-care systems requires promoting autonomy and transferring power and authority to local communities and health-care consumers. In such case, Laura Alston and colleague call for investment in place-based research in small rural hospitals, where local clinicians have a deeper understandings of community health needs, to address health disparities in rural Australia. Earlier empirical evidence of innovation implementation in small-scale, real-world settings, as highlighted in the *Innovation in the Western Pacific* special collection, shows the potential and impact of engaging and empowering the target population to optimise health outcomes. These lessons must be grounded in theory and tested across diverse contexts and populations to benefit a wider population.

Given the resource-intensive nature of medical services and financial constraints in most countries of the region, digital health has rapidly evolved to address complex health challenges and disparities. Other notable trends in the Asia–Pacific region include the growing number of oncology clinical trials offering new prevention and treatment options in the region and globally, and the increasing influence of Asia–Pacific countries on global health, which has potential to drive the global health agenda.

Looking ahead, there is much to anticipate. The upcoming Series on low birth rate will explore the social and biological drivers of low fertility in the Asia–Pacific region. The *Child and Adolescent Mental Health in the Western Pacific Series* emphasises the importance of political commitment and investment to build comprehensive, evidence-based mental health systems for children and adolescents. We will also publish a joint Series on *Mature T-cell and Natural Killer-cell Lymphomas* with *The Lancet Haematology*, reflecting our focus on new treatments and interventions for cancer and other prevalent NCDs in the region. As stated in our inaugural issue, we aspire “to be a leading and open forum for constructive dialogue among health practitioners, communities, and policymakers,” to better address local health needs and ensure that the voices and stories of the Western Pacific region are amplified.

